# Recent advances in pericardium extracellular matrix for tissue regeneration, along with a short insight into artificial intelligence

**DOI:** 10.3389/fmedt.2025.1503153

**Published:** 2025-08-14

**Authors:** Parand Shariat Rad, Mozafar Khazaei, Elham Ghanbari, Mehdi Rashidi, Leila Rezakhani

**Affiliations:** ^1^ Student Research Committee, Kermanshah University of Medical Sciences, Kermanshah, Iran; ^2^ USERN Office, Kermanshah University of Medical Sciences, Kermanshah, Iran; ^3^ Fertility and Infertility Research Center, Health Technology Institute, Kermanshah University of Medical Sciences, Kermanshah, Iran; ^4^ Department of Tissue Engineering, School of Medicine, Kermanshah University of Medical Sciences, Kermanshah, Iran; ^5^ Department of Mathematics and Physics “E. De Giorgi”, University of Salento, Lecce, Italy

**Keywords:** pericardium, decellularization, regeneration, extracellular matrix, machine learning

## Abstract

Medical science is striving to find new solutions to treat various diseases. Tissue engineering with a great potential to develop tissues and even organs from synthetic and biological materials, open a new gate toward absolute treatments. Although in tissue engineering as a subtype of regenerative medicine, decellularized tissues are new, promising way to fill the previous methods gaps. Outside of the biological aspects, artificial intelligence (AI) and machine learning (ML) are applied to tissue engineering. Decellularization is a very important area where AI supports protocols and ensures the process is repeated identically each time. It also greatly assists in monitoring the extracellular matrix (ECM) to ensure it remains intact. Nonetheless, the use of AI in tissue engineering is not fully discussed in scientific articles. Although based on the tissue used for decellularization these features could vary, to optimize decellularization we need new method to reach high accuracy. In these current days, Pericardium, a double-layered membrane around the heart of mammalians, as a natural ECM has been utilized in cardiac surgery for many years. However, the use of decellularized pericardium as a scaffold for tissue engineering has gained significant attention in recent times, due to its retention strength, flexibility, supports for cell growth and differentiation, etc. That altogether put it among the top choices for tissue engineering and regenerative medicine. In this review we aim to cover the different decellularization methods, application of decellularized pericardium, commercial products that are available and challenges and future direction of this potent therapy.

## 1 Introduction

Tissue engineering has recently become a ground-breaking area of regenerative medicine, offering patients with organ failure or tissue damage fresh hope. The potential for transforming healthcare and enhancing patient outcomes is enormous when it comes to the ability to grow functional tissues and organs in the lab. Utilizing decellularized tissues like pericardium is one of the promising strategies in tissue engineering. To construct functional tissues, tissue engineering combines scaffolds, cells, and chemical agents with physiological activity. It emerged from the development of biomaterials. Tissue engineering aims to produce functional structures that restore, protect, or improve injured tissues or complete organs (1–6).

In 2021, Barbolescu and et al, given that gold standard methods for scaffold evaluation are lacking and scaffold destruction is required, developed an artificial intelligence-based method using deep convolutional neural networks (DCNN) to accurately identify the different stages of decellularization, which allows for precise determination of the completion time of the process. Apart from the tissue engineering approach, ML and deep learning can support increasing the quality of histological analysis and DNA removal (7).

The pericardium is a thin, double-layered membrane that cover the heart, and has special qualities that make it a prime target for tissue engineering. The ECM remains after decellularization procedure, and it keeps its structural integrity and bioactive molecules while removing immunogenicity and potential dangers of disease transmission (8–10). When decellularized and employed as a hydrogel, the decellularized pericardium can generate a three-dimensional environment that promotes tissue growth. As a bioink, it provides a customized platform for three-dimensional (3D) bioprinting, enabling the exact deposition (11) of cells and ECM to replicate genuine tissue architecture. Also, electrospinning decellularized pericardium can create fibrous scaffolds that mimic the ECM's nanofibrous structure, offering a large surface area for cell contact and nutrition exchange. These applications demonstrate the potential of decellularized pericardium in tissue engineering and regenerative therapy (8, 12, 13).

Comparing decellularized pericardium to synthetic materials, which are frequently utilized in tissue engineering, reveals significant advantages. Because of the near resemblance between its natural makeup and that of native tissues, it offers the ideal milieu for cell adhesion, proliferation, and differentiation. It also possesses superior mechanical qualities including tensile strength and elasticity, which guarantee the stability and functionality of designed tissues (14, 15). The use of decellularized pericardium in tissue engineering has produced encouraging outcomes in several domains. Researchers have successfully used this biomaterial to create viable replacements for harmed or diseased tissues in a variety of fields, from orthopedics to cardiovascular regeneration. Scientists can direct cellular behavior, improve tissue regeneration, and minimize immunological rejection by seeding patient-specific cells onto the decellularized scaffold (8, 16–19).

**Figure 1:**
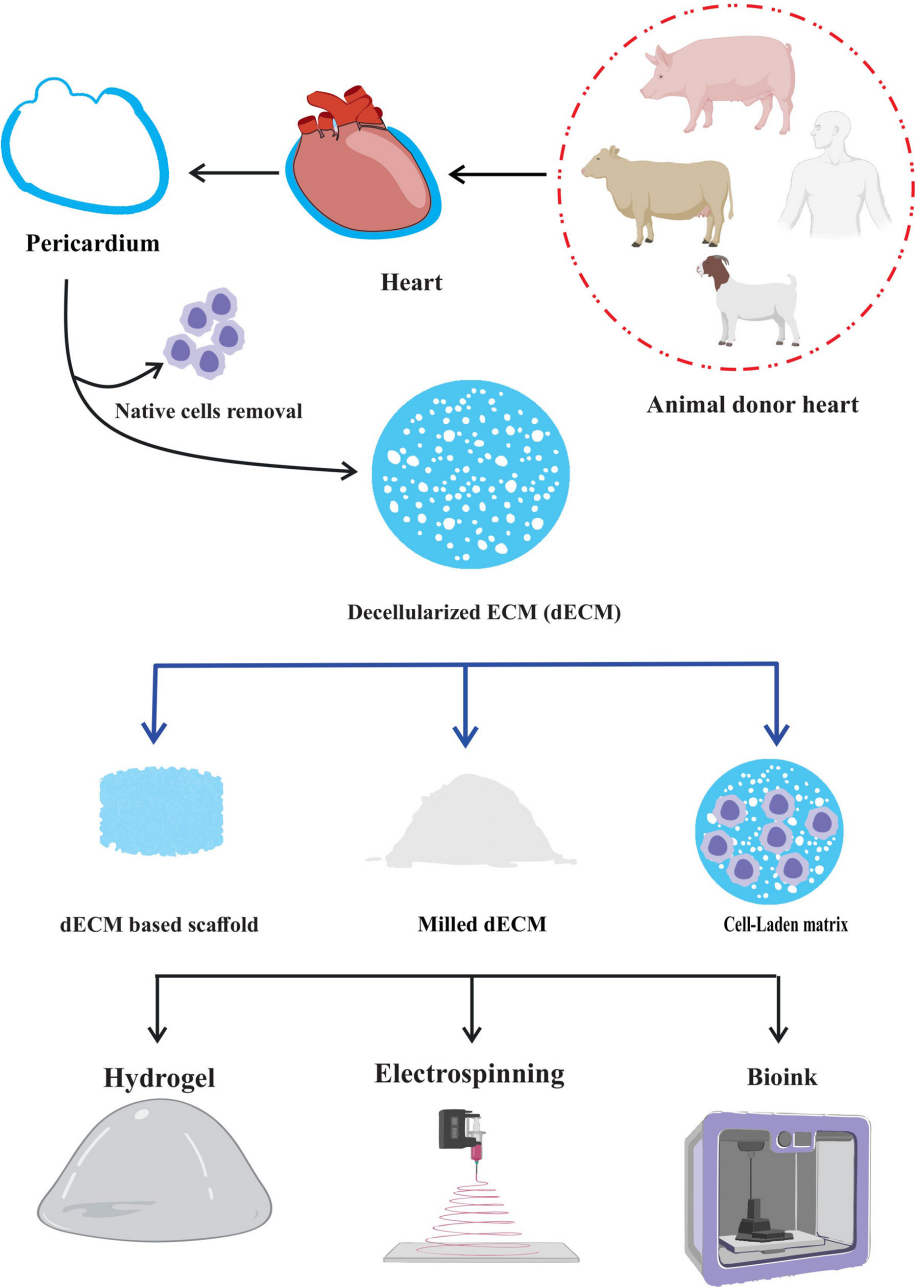
Decellularized pericardium and its application.

Decellularized pericardium can also be easily modified to suit the needs of other tissues or organs. To improve its mechanical qualities, it can be molded into certain geometries, mixed with other biomaterials, or added growth factors for specific biological responses. Due of its versatility, it can be used to build intricate, 3D structures that closely resemble the structure of natural tissues (20–22). The use of decellularized pericardium has enormous promise for tackling important problems in regenerative medicine as research in this area develops. This cutting-edge strategy offers a workable answer for individualized treatment and better patient care, from replacing broken heart valves to mending bone deformities or even creating bioartificial organs (23–25). Here, we discuss the most cutting-edge methods for decellularizing pericardium and highlight recent developments in their use in a variety of tissue engineering disciplines and limitation of decellularization method in this era. Understanding this method's possible advantages and disadvantages may help us develop more potent treatments that make use of nature's own building blocks for repairing damaged tissues and regaining normal physiological function (Figure 1).

## 2 Different pericardium decellularization approaches

In numerous investigations, several pericardium decellularization techniques have been investigated. A perfusion-assisted bioreactor system employing pig pericardium as the tissue source is one such technique. In an *in vitro* model research, the pericardium underwent a decellularization treatment that included 24 h at room temperature (RT) in 0.1% (w/v) sodium dodecyl sulphate (SDS) and 24 h in 1% (v/v) Triton X-100 (TX) (19). Overall, SDS and TX are the most common materials used for decellularization. But the point is about the timings, temperature, shaker round per minute, washing methods, etc. that this process take place (26, 27).

Another study used an *in vitro* cell culture model to treat porcine pericardium with SDS. The decellularization procedure involves treating the tissue for 24 h with 0.5% SDS and 0.5% SDC after exposing it to high hydrostatic pressure of 1,000 MPa at 30 °C for 10 min using a high-hydrostatic pressure machine (15). *In vivo* research using human participants and swine pericardium as the tissue source has been done to investigate the effects of PLA2 and sodium deoxycholate (SD) treatment combined. The pericardium was treated with 200 U/ml PLA2 and 0.5% (w/v) SD as part of the decellularization process, which took place over the course of 2 h at RT with constant shaking (20). High hydrostatic pressure is another potential technique, uses to decellularized rat pericardium in a study, have present special features. A cold isostatic pressure machine was used to subject the pericardium to a high-hydrostatic pressure of 980 MPa for 10 min at 30 °C in an *in vivo* rat model study (28). Another decellularization procedure that has been investigated for swine pericardium is hypotonic and hypertonic rinses that is used with deionized water (dH2O) and subsequent treatment with 1% SDS in phosphate-buffered saline (PBS) for 24 h, for both *in vitro* and *in vivo* experiments (29).

TRITDOC (TRIton-X100 and TauroDeOxyCholic acid) decellularization procedure, introduced as a holistic approach to heart valve tissue engineering (30) which has evaluation during time (31). In this procedure Pericardial patches were treated with alternating hypo- or hypertonic solutions, TX (0.1%–1%), and 4 mM sodium taurodeoxycholate. The extractions were performed in a degassed solution with 10 mM sodium ascorbate and 5 mM ethylenediaminetetraacetic acid (EDTA) under N2 atmosphere and continuous stirring. The residual nucleic acids were digested with 1,500 U cm^−2^ of the non-specific endonuclease Benzonase™ at 37 °C for 24 h. The solutions were sterilized using Filtropur filters with a pore size of 0.20 μm. TBP was decellularized and kept at 4 °C in an antibiotic/antimycotic solution containing 3 U ml^−1^ penicillin, 3 mg ml^−1^ streptomycin, and 2.5 μg ml^−1^ Amphotericin B (30–32).

**Figure 2:**
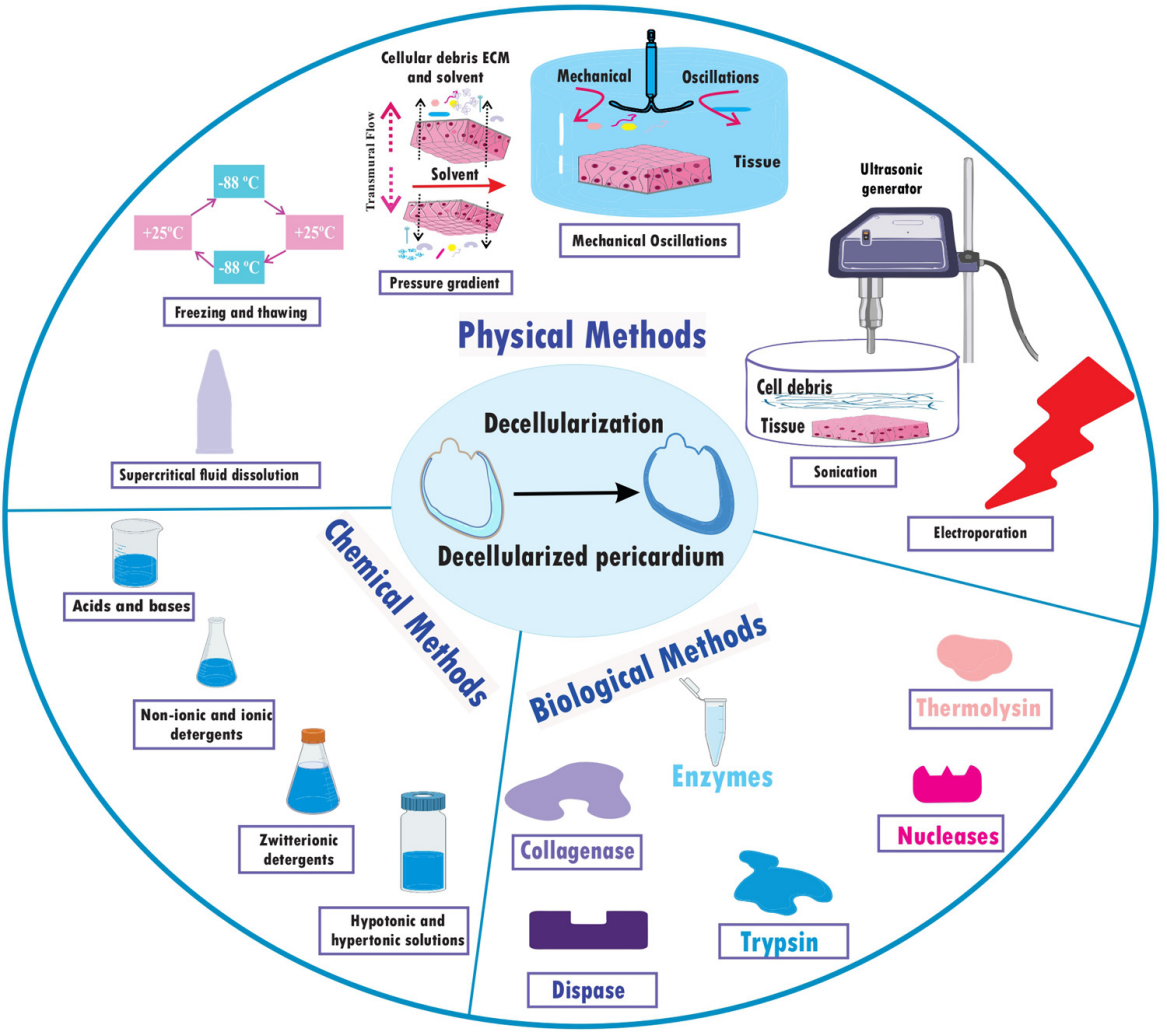
Decellularization approaches; physical, chemical, and biological methods.

A fixative-free decellularization technique has been examined in an *in vivo* investigation employing swine pericardium as the tissue source, including participants from both the pig and human species. This procedure made use of a hypotonic (10 mM Tris-HCl; pH 8.0) detergent solution containing 0.1% (w/v) SDS and 50 U ml^−1^ DNase-I and 1 U ml^−1^ RNase-A, and it doesn’t glutaraldehyde (GA) as frequency used fixative for histological processes (33). Finally, non-chemical methods of decellularization that are non-toxic and may work faster could provide a suitable mechanism for decellularization. Freeze-thawing is one of the methods which shows to be successful. Freezing and thawing tissue for 20–30 cycles, each lasting 2–5 min was enough to cause noticeable tissue decellularization (34). These numerous techniques give multiple ways to successfully decellularize pericardial tissues from various animal sources, and they can offer insightful information for creating tactics that will work for upcoming application (26) (Table 1) (Figure 2).

**Table 1:** Different decellularized approaches.

Pericardium origin	Model study	Decellularization approach	Ref.
Pig	Bioreactor system (*in vitro*)	- 24 h in 0.1% SDS at RT - 24 h in 1% TX at RT	(19)
Rat	Rat (*In vivo*)	High-hydrostatic pressure of 980 MPa for 10 min at 30 °C by a cold isostatic pressure machine	(16)
Bovine	Rat (*In vivo*)	20 ml of SDS on a heated plate shaker at 37 °C at 70 rpm for 7 h	(23)
Porcine	Human (*In vivo*)	200 U/ml PLA2 and 0.5% (w/v) SD under continuous shaking for 2 h at RT	(28)
Bovine	*In vitro*	TRITDOC decellularization procedure	(32)
Porcine	*In vitro*	High hydrostatic pressure of 1,000 MPa at 30 °C for 10 min using a high-hydrostatic-pressure machine_ 0.5% SDS and 0.5% SDC for 24 h	(15)
Porcine	*In vitro*	1% SDS solution for 2h	(35)
Porcine	*In vitro*	0.025% TS diluted in phosphate buffered saline for 24 h at 37 °C	(36)
	Swine (*In vivo*)	1% TX and 0.1% ammonium hydroxide for 72 h at 4 °C.	
Bovine	Sheep (*In vivo*)	GA (Partial cross-linking was assessed by estimating fixation index using Ninhydrin assay following 24 h digestion with type 2 collagenase)	(29)
Sheep	*In vitro*	Freeze-thaw method for 20 and 30 cycles with 2-min freezing and 2 or 5-min thawing	(34)
Porcine	*In vitro*	Hypotonic and hypertonic rinses in dH2O and 1% SDS for 24 h	(37)
	Rat (*In vivo*)		
Human	*In vitro*	1% SDS for 60–65 h	(37)
	Rat (*In vivo*)		
Pig	human and pig (*In vivo*)	0.1% SDS and 50 U ml^−1^ DNase-I and 1 U ml^−1^ RNase-A	(38)
Porcine	Porcine (*In vivo*)	SDS and DNase and dH2O	(39)
Human	*In vitro*	0.1% SDS, 7 mM EDTA, for 24 h at RT, washed with 70% ethanol for 24 h at RT followed by 10 days of washing with 0.9% NaCl	(8)
Human	*In vitro*	Hypertonic solution and two reagents (benzonase and sodium cholate)	(18)
Rabbit	Rabbit (*In vivo*)	0.5% SDS for 24 h	(40)
Bovine	*In vitro*	1% SDS	(41)
		1% SDS + 0.5% SD	
		1% TX,	
		1% TX + 0.5% SD	
		freeze–thaw cycles + 1% SDS + 0.5% SD freeze–thaw cycles + 1% TX + 0.5% SD	
Bovine	Donkey *(In vivo*)	Ethanol combination (4%) and 0.1% peracetic acid for 2 h	(42)
Bovine	*In vitro*	Hypotonic solution for 36 h at 4 °C followed by a 24 h rinse in a 1% TX, DNase/RNase solution for 1 h in 37 °C, again for 24 h in 1% TX at RT	(43)
ovine	*In vitro*	0.05 SDS and 0.05% SD for 24 h	(44)
Bovine	*In vitro*	Hypotonic and subsequently hypertonic Tris	(45)
	Mice (*In vivo*)	with 0.02% EDTA at 4 °C for 24 h	
		agitated in Tris-HCl with 1% TX	
		and 0.02% EDTA at 4 °C for 24 h	
		Incubated with 20 mg/ml RNase A and 0.2 mg/ml DNase I at 37 °C for 2 h	
		Tris-HCl with 1% TX at 4 °C for 24 h	
Porcine and Ovine	*In vitro*	SDS or DNase, with dH2O	(46)
Bovine	*In vitro*	SDS 1% for 48 h at 40 °C then Vacuuming	(47)
Porcine	human *(In vivo*)	0.1% EDTA and 0.1% SDS for 24 h while shaking at RT	(48)
Pig	*In vitro*	10 mM Tris-HCl; pH 8.0	(38)
		0.1% SDS	
		50 U ml^−1^ DNase I and 1 U ml^−1^ RNase A	
Porcine	*In vitro*	0.5% TX for 24 h, followed by 0.5% SDS for 24 h at RT	(49)
Bovine	*In vitro*	DDE group: hypotonic and hypertonic Tris-HCL with 0.02% EDTA at 48 °C for 24 h. then agitated in Tris–HCl with 1% octylphenoxypolyethanol and 0.02% EDTA at 48 °C for 24 h. incubation with 20 mg/ml RNase A and 0.2 mg/ml DNase I at 37 °C for 2 h. then placed in Tris–HCl with 1% TX at 48 °C for 24 h.	(50)
		TS group: 24 h in a solution containing 1% TS, 0.02% EDTA, RNase A (20 mg/ml), and DNase I (0.2 mg/ml) at 37 °C.	
		TSD group: 24 h in a solution containing 0.5% TX, 0.5% TSD, 0.02% EDTA, RNase A (20 mg/ml), and DNase I (0.2 mg/ml) at 37 °C. Then digested for 48 h in a solution contained 0.25% TX, 0.25% TSD, 0.02% EDTA, RNase A (20 mg/ml), and DNase I (0.2 mg/ml) at 37 °C	
Bovine	*In vitro*	1% SDS for 48 h at 40 °C	(51)
Bovine	*In vitro*	(a) 1 M NaCl, 8 mM CHAPS detergent and 25 mM EDTA	(52)
		(b) 1 M NaCl, 1,8 mM SDS and 25 mM EDTA.	
		(c) 6.4 μM DNase I from bovine pancreas, 0.1 M MgCl and 0.9 M NaCl	
Steer	*In vitro*	hypotonic solution for 36 h at 48 °C, then for 1 h exposed to RNase and DNase, finally 24 h in a 1% TX solution at RT	(53)
Bovine	*In vitro*	TRICOL protocol [hypo- and hypertonic solutions, 0.1%–1% (w/v) TX, and 10 mM sodium cholate detergents]	(54)
Bovine	*In vitro*	freeze–thawed (80 °C for 4 h followed by 37 °C for 30 min) three times, then treated with a mixture of 0.25% TX and 1% SD for 24 h. finally incubated in a mixture of DNase (200 mg/ml) and RNase (150 lg/ml) at 37 °C for 1 h.	(55)
	Rabbit (*In vivo*)		
Human	*In vitro*	Treated with D.D.W. for 2 h, 1% SDS for 24 h, washed in RNase/DNase nuclease solution, and finally treated with acetic acid (0.2M).	(56)
Bovine	*In vitro*	1% TX in the tris-buffered salt solution (10 m mol/L, pH8.0) with protease inhibition for 24 h at 4 °C then it digested with DNase (50 U/ml) and RNase (1 U/ml) at 37 °C for 1 h.	(57)
Bovine	*In vitro*	0.5% TX and 0.5% SD for 24 h at 37 °C	(58)
Porcine	Mouse (*In vivo*)	0.1% TX solutions with 0.1% pepsin and ultrasonically digested under 30 kHz for 48 h	(59)
Porcine	*In vitro*	0.1% EDTA acid and 0.1% SDS for 24 h	(48)
Bovine	*In vitro*	1% TX, 0.1% SDS, 150 mM NaCl and 1% deoxycholic acid in 10 mM Tris-HCL buffer (pH 7.4) with protease inhibitor, for 12 h at 4 °C	(60)
Human	Swine (*In vivo*)	1% SDS for 72 h and then in 1% TX for 48 h	(61)
Human	*In vitro*	0.1% SDS for 24 h and then in RNase/DNase solution for 24 h	(62)
Swine	*In vitro*	0.25% SD, 0.15% TX, 0.1% EDTA, 0.02% NaN3, in 50 mM Tris-HCL buffer (pH 7.8) for 6 days at 22 °C	(63)

## 3 Mechanical properties

**Figure 3:**
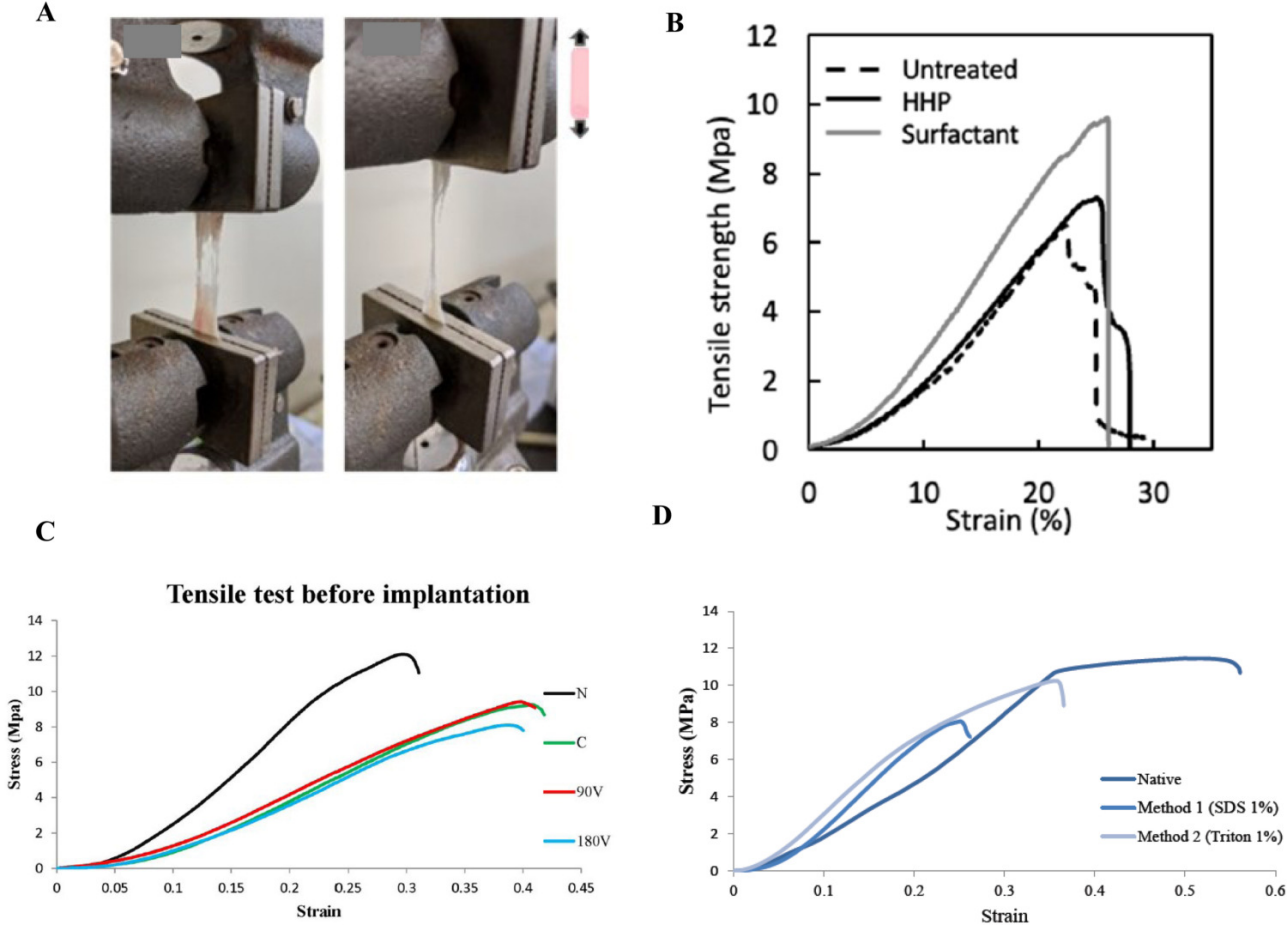
Mechanical test, **(A)** shows how we access and measure the tensile strength, **(B)** (15), depicts typical stress-strain curves for the untreated pericardium, high hydrostatic pressurization (HHP)-decellularized pericardium, and surfactant-decellularized pericardium, **(C)** (49) shows strain measurements of vacuumed decellularized pericardium before and after implantation, and **(D)** (82) illustrates the stress-strain curves for decellularized tissue in native, SDS-treated, and TX-treated pericardium. Various studies show that tensile strength (Young's modulus) decreases in decellularized tissues compared to native tissue. The type of detergent and the decellularization method have a significant effect on reducing the mechanical properties. As can be seen, the HHP method and the use of surfactants have a higher Young's modulus than the SDS method.

In tissue engineering applications, the mechanical characteristics of decellularized tissues are extremely important. Several significant characteristics of decellularized pericardium have been identified. And here we’re going to discuss some of the most effected ones during decellularization approaches. Tensile Strength and Modulus of Elasticity (Young's modulus) (Figure 3).

Tensile strength gauges a material's capacity to withstand pulling or stretching forces without breaking. The decellularized pericardium's capacity to preserve structural integrity under tension is determined by its tensile strength (39). The elastic modulus, sometimes referred to as Young's modulus, gauges a material's stiffness or rigidity. It explains how much a material deforms when a force is applied and how much it straightens out again when the force is removed. Decellularized pericardium's elastic modulus is an indicator of its capacity to withstand mechanical stress and keep its shape (19).

The impact of decellularization on tensile strength and elasticity has been investigated in various research; While the general trend suggests that decellularization does not significantly affect the ultimate tensile strength compared to native tissue (65, 66). Here are some specific findings and comparisons that can show the alternations. Studies comparing decellularized and glutaraldehyde-fixed pericardium reveal distinct differences. GA fixation generally increases stiffness and reduces elasticity. The following data illustrate that while GA treatment results in higher tensile strength, it also leads to significantly higher elongation at break, indicating reduced elasticity.

Mildly Cross-linked Decellularized Bovine Pericardium (29):

Tensile Strength: 13.14 ± 4.32 MPa
% Strain at Max Load: 47.11 ± 20.4%
Young's Modulus: 50.08 ± 18.9 MPa
Elongation at Break: 61.2 ± 19.7%

Commercially Available Glutaraldehyde-Treated Bovine Pericardium (29):

Tensile Strength: 16.7 ± 6.3 MPa
% Strain at Max Load: 71.19 ± 7.8%
Young's Modulus: 47.46 ± 15.76 MPa
Elongation at Break: 86.33 ± 9.03%

In another study they conduct direct comparisons between native and decellularized pericardium, often shows no statistically significant differences in tensile strength or elastic modulus. As the following data confirm, the differences in the numbers were not significant (*P* value >0.05), supporting the theory that decellularization can preserve the native mechanical integrity. Studies on porcine pericardium also confirm these findings.

Native Bovine Pericardium (NBP) (32):

Elastic Modulus: 51.4 ± 4.6 MPa
Ultimate Tensile Strength: 17.3 ± 0.8 MPa

Decellularized Bovine Pericardium (TBP) (32):

Elastic Modulus: 48.7 ± 4.9 MPa
Ultimate Tensile Strength: 15.3 ± 1.2 MPa

Another important aspect in this section is the impact of decellularization Methods; Different decellularization protocols can have varying effects. While some studies using high hydrostatic pressure or surfactant methods; they found no significant differences in ultimate tensile strength, failure strain, and elastic modulus, and even others observed changes in Young's modulus depending on the specific detergent (e.g., SDS) concentration. One study on porcine pericardium treated with SDS showed the following (35):

Native Pericardium: Young's moduli of 36.23 ± 3.2 MPa
0.5% SDS: Young's moduli of 13.66 ± 0.9 MPa and 26.81 ± 3.8 MPa
1% SDS: Young's moduli of 12.17 ± 5.5 MPa and 36.26 ± 2.9 MPa

So, these results suggest that SDS treatment can influence the elastic modulus (increase in SDS concentration decrease the elasticity), although the differences were not always statistically significant.

Other mechanical characteristics:

**Distensibility** is an important mechanical property of tissues, which researcher for evaluating their works, assess it through methods like confined-flow perfusion, biaxial inflation, and pressure-volume curves. These curves could provide insights into tissue stiffness, and to make their interpretations easy, you should know that higher slopes indicating increased resistance to deformation. But pressure–volume (P–V) curve analysis goes further than this. In most of the studies decellularized pericardium shows similar behavior to native tissue, while GA-treated tissue exhibits higher curve slopes, which as explained present as having a significantly higher tissue stiffness of both elastin and collagen fibers. While decellularization can reduce GAG content and potentially increase thickness, its overall impact on distensibility varies depending on the specific method and the tissue source. As you noticed, understanding these factors is critical for optimizing tissue engineering strategies and developing biocompatible pericardial grafts (38, 67, 68).
**Permeability** is defined as the ability of a material to allow fluids to pass through. It's a factor that play a role in the success of pericardial tissue engineering. Various methods, including perfusion tests and molecular transport studies, can be used to test this property of our product. Decellularization procedures, particularly TX, enhance permeability significantly by disrupting cell-matrix interactions, this helps cell seeding, nutrient/oxygen transport, and overall recellularization efficiency. Increased thickness can also contribute to higher permeability. Maintaining adequate mechanical strength and preventing excessive fluid flow remain should always be considered for successful tissue engineering applications (19, 23, 69).
**Stress-Strain Behavior** is another property for decellularized tissue which is tested by Uniaxial Tensile Loading (UTL) Test. Decellularized pericardium typically demonstrate a J-shaped stress-strain curve. These curves were generated to determine the biomechanical properties. The elastic modulus, representing the slope of the stress-strain curve, was measured at low (E1) and high (E2) strain (E1 reflects tissue resistance due to elastin fibers, while E2 reflects resistance from collagen fibers). Based on the study and final application of decellularized tissue, this test may be necessary (8, 19, 24).
**Suture retention strength** is a crucial factor for successful surgical grafts. It's being evaluated in a composite material made of decellularized ECM and a synthetic polymer (the maximum force required to pull a suture through the material). dECM appears to have significantly greater suture retention strength than native tissues (independent of the polymer's crosslinking method). Although the retention decreased with enzymatic degradation of the matrix, highlighting the importance of the decellularized matrix component and the method used for decellularization. This superior strength suggests a reduced risk of graft failure at the surgical connection site, reveal as a critical advantage for *in vivo* applications (23, 44).
**Circumferential stress**, a measure of a vessel's resistance to outward pressure. In a study worked on a decellularized porcine right coronary artery, it evaluated in ring-shaped dECM + PPF grafts using mechanical testing. Rather than the addition of the synthetic polymer (PPF) greatly dictated the graft's circumferential strength in this study, increasing UV exposure time leads to an increase in stress resistance (from 2.24 ± 0.99 MPa to 4.20 ± 2.05 MPa between 15 and 45 min). Notably, enzymatic degradation, mimicking *in vivo* breakdown, led to increased circumferential strength, likely due to the inherent properties of the PPF. This suggests that the dECM contributes to the graft's mechanical integrity even after some degradation (38, 70).

According to these, Decellularization effectively preserves many key biomechanical characteristics, while it also greatly depends on decellularization method and source of the tissue.

## 4 Application of decellularized pericardium in regeneration

In numerous models and applications, the decellularized pericardium has demonstrated encouraging potential for tissue regeneration. A perfusion-assisted bioreactor system was used to regenerate the aortic valve in a model study utilizing pig pericardium. Enhanced cell seeding with uniform distribution and aortic valve cell adhesion to decellularized scaffolds were the main findings of this investigation. The seeds showed functional traits typical of mature heart valve cells and persisted in viability. Additionally, the planted cells displayed enhanced ECM integration, suggesting potential for tissue remodeling and regeneration. The synthetic structures also showed mechanical characteristics like those of natural heart valves, indicating feasibility for implantation in the future (19). The goal of a different study that used rat pericardium was to prevent cardiac adhesion. In the rat model, the application of fibrin-coated pericardial ECM successfully prevented heart adhesion. In addition, when compared to control groups that received no ECM therapy, this strategy promoted tissue regeneration and decreased scar formation (28). Research into tissue regeneration in the pulmonary artery using equine pericardium has been conducted. Positive results were seen in the first single-center trial with Matrix PatchTM, which involved 10 patients. Improvements in hemodynamics and a lack of graft-related problems such thrombosis or infection were seen. During the follow-up period, no indications of rejection or harmful immunological reactions were found (37).

It has been investigated to create small-diameter vascular grafts using pericardium from cows. Pericardial tissue was successfully decellularized while maintaining the content and structure of the ECM. Rat smooth muscle cells (SMCs) exhibited increased adhesion and proliferation in *in vitro* experiments when grown on a decellularized pericardial ECM scaffold, demonstrating biocompatibility. Studies conducted *in vivo* further confirmed its promise as a small-diameter vascular graft, demonstrating high patency and tissue integration, low thrombogenicity, and little inflammatory reaction (32). In an *in vitro* experiment using human conjunctival epithelial cells and fibroblasts, crosslinked decellularized swine pericardium demonstrated promise in promoting cell adhesion, proliferation, and differentiation. The substance showed excellent biocompatibility and had no harmful effects on the test cells. Additionally, it encouraged cell migration and the development of multilayered epithelial structures that resembled natural conjunctival tissue (20).

The interaction between human umbilical vein endothelial cells (HUVECs) with both decellularized bovine pericardial patches and commercial bioprosthetic heart valves was examined in an *in vitro* comparative study employing bovine pericardium. The two materials’ mechanical attributes, including tensile strength and elasticity, were contrasted. Additionally, to assess the likelihood of immunological reactions, an analysis of the immune response elicited by both materials was done (23). In an *in vitro* cell culture model, the porcine pericardium has also been investigated for ligament-like tissue regeneration. Cell survival, proliferation, and ECM production in fibroblasts grown on a scaffold made from decellularized pericardium were all good. The regenerated tissue displayed traits, such as collagen synthesis and alignment, that were comparable to those of natural ligaments. Mechanical testing revealed that the tissue's strength was on par with that of native ligaments (15).

The decellularization procedure successfully eliminated cellular components while retaining the ECM structure in an *in vitro* cell culture investigation utilizing swine pericardium. Compared to other experiments, HUVECs successfully attached and created functional endothelium monolayers on the scaffold surface in a short amount of time. Human mesenchymal stem cells (hMSCs) also showed development into cells that resembled smooth muscles, suggesting the possibility of tissue regeneration (35). Another study found that a porcine pericardium-based nanomaterial-tissue patch had promising mechanical properties suited for cardiovascular applications. The patch, maintained cell adhesion, proliferation, and survival with success *in vitro*, indicating its viability as a viable treatment for and vascular repair (36).

An experiment employing an animal model and bovine pericardium revealed long-term biocompatibility with little inflammatory reaction even after long-term implantation. The patch shape facilitated fresh ECM deposition by host cells as well as incremental host cell infiltration and repopulation over time, indicating tissue regeneration. The patches exhibited long-term stability and endurance throughout the study period, maintaining their structural integrity and functionality (38). The physical, mechanical, biocompatibility, and angiogenesis-promoting characteristics of an injectable pericardial matrix gel were studied to create an injectable scaffold that could assist heart tissue regeneration. When injected into rat hearts, the gel demonstrated good biocompatibility and encouraged the growth of new blood vessels, demonstrating its potential to enhance tissue regeneration (29). According to a study that used decellularized pig pericardium as a scaffold for aortic valve interstitial cells (VICs), both pig and human VICs demonstrated good viability, proliferation, and adhesion. This implies that the fixative-free decellularization technique efficiently eliminates cellular components while maintaining the integrity of the ECM, enabling successful cell seeding (16).

**Figure 4:**
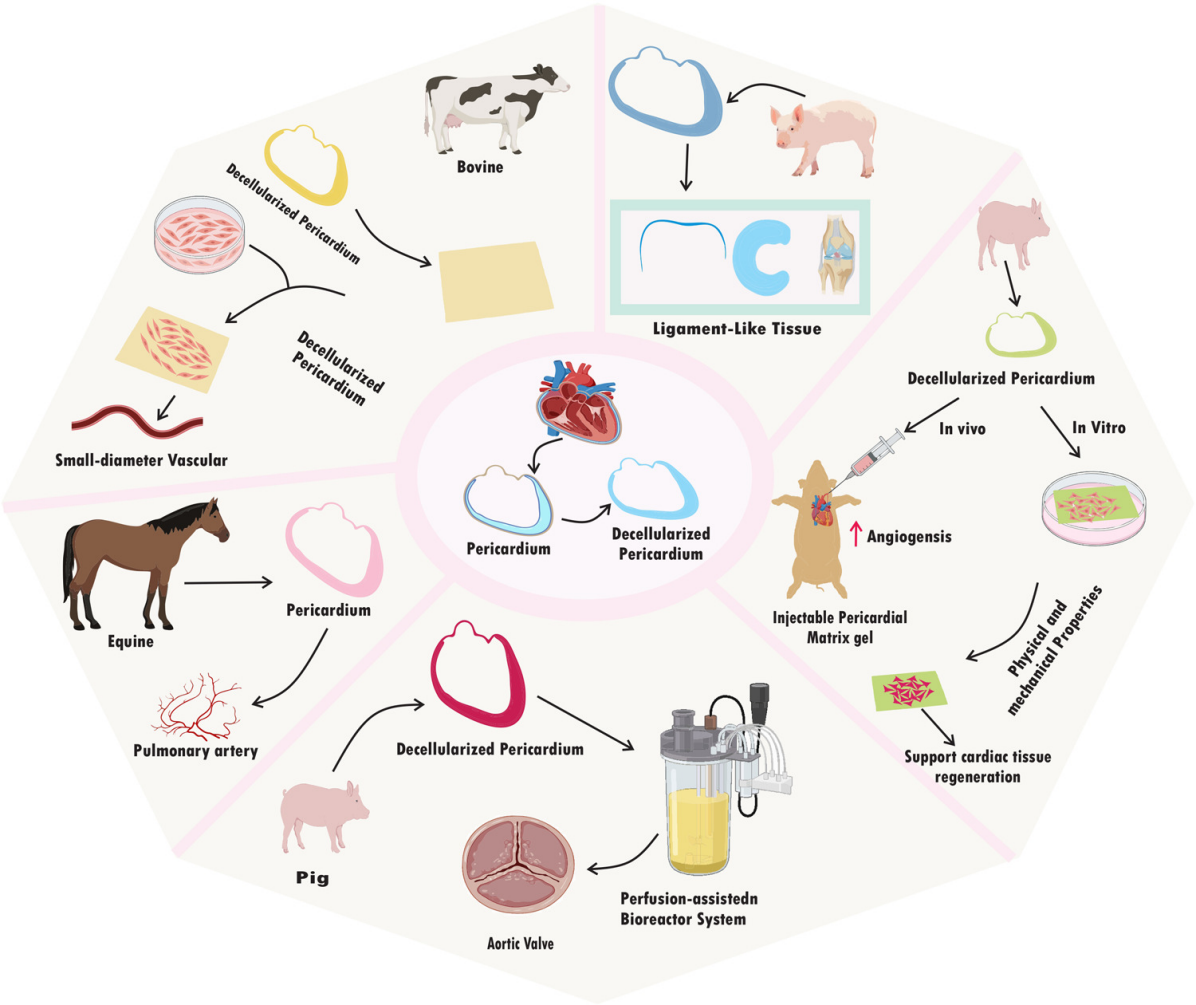
Application of decellularized pericardium.

The effective recellularization of decellularized pericardial scaffolds with autologous or allogeneic cells was also seen in a pig carotid artery model, as well as signs of vascular remodeling in response to recellularized patches and matrices. These results imply a great potential for decellularized pericardium to be used in tissue regeneration, particularly in the setting of blood vessels and cardiac tissue (39). All things considered, this research show the various uses of decellularized pericardium in tissue regeneration across various models, tissues, and organs. The research shows that it has the potential to improve cell seeding, promote cell viability/functionality/integration with ECM, maintain mechanical properties like native tissue, prevent adhesion and scar formation, improve hemodynamics, support cell adhesion/proliferation/differentiation, promote tissue regeneration and remodeling, and exhibit biocompatibility with minimal immune response. Decellularized pericardium has the potential to be a flexible biomaterial for a range of regenerative medicine applications, such as ligament-like tissue regeneration, vascular graft creation, conjunctival tissue repair, and more. To fully explore its potential and maximize its usage in clinical settings, additional study in both the laboratory and the clinic is required (71) (Table 2) (Figure 4).

**Table 2:** Application of decellularized pericardium in regeneration and its outcomes.

Product name	Model study	Tissue regeneration	Major outcomes	Ref.
Pig	Bioreactor system (*in vitro*)	Aortic valve	- Enhanced cell seeding - Cell viability and functionality - Improved tissue integration - Comparable mechanical properties to native heart valves	(19)
Rat	Rat (*In vivo*)	Prevent heart adhesion (no regeneration)	- prevented heart adhesion - facilitate tissue regeneration - reduced scar formation	(16)
Equine	Human (*In vivo*)	Pulmonary artery	- Improved hemodynamics, - absence of graft-related complications such as thrombosis or infection - no signs of rejection or adverse immune reactions	(20)
Bovine	Rat (*In vivo*)	Developing small-diameter vascular grafts	- Enhanced adhesion and proliferation of SMCs - Good patency (openness) and integration of graft with host tissue - Low thrombogenicity and minimal inflammatory response	(23)
Porcine	Human (*In vivo*)	Conjunctival epithelial cells and fibroblasts	- Supported cell adhesion, proliferation, and differentiation - Good biocompatibility with no cytotoxic effect - Promote cell migration and formation	(28)
Bovine	*In vitro*	Bioprosthetic heart valves	- Biocompatibility assessment - Comparable mechanical properties - Immunogenicity evaluation	(32, 72–74)
Porcine	*In vitro*	Ligament-like tissue	- Good cell viability, proliferation, and ECM synthesis - The regenerated tissue exhibited characteristics similar to native tissue	(15)
Porcine	*In vitro*	Blood vessel	- Successful adhesion and formation of functional tissue on the scaffold surface - Indicating the potential for tissue regeneration	(35)
Porcine	*In vitro*	vascular and cardiac tissue	- Supports cell adhesion, proliferation, and viability - Promising suitable mechanical properties	(36)
	Swine (*In vivo*)			
Bovine	Sheep (*In vivo*)	Ascending aorta	- Long-term biocompatibility - Cell infiltration and repopulation - ECM deposition - Mechanical properties improved over time - Long-term stability	(29)
Porcine	*In vitro*	cardiac tissue regeneration	- Suitable physical and mechanical properties of gel - Attachment and proliferation without cytotoxicity - Good biocompatibility and no signs of inflammation or adverse reactions - Promote angiogenesis	(37)
	rat (*In vivo*)			
Pig	human and pig (*In vivo*)	Aortic valve	- Good viability, proliferation, and attachment - Successful cell seeding	(38, 75–77)
Porcine	Porcine (*In vivo*)	Vessels	- Effective recellularization - Vascular remodeling - Comparable results between autologous and allogeneic cell sources. - Enhanced mechanical properties	(39)
Bovine	Donkey (*In vivo*)	Donkeys’ distal limb injuries	- faster healing process - cost-effective	(42)
Ovine	*In vitro*	Skin tissue engineering	- good properties for cell seeding - easy to decellularize - capability of vascularization - no significant toxicity and MTT test	(64)
Sheep	Mice (*In vivo*)	mice wound	- enhanced skin wound healing by suppressing proinflammatory mediators - promoting cell growth, angiogenesis, and the formation of granulation tissue by VEGF	(78)
Bovine	Human (*In vivo*)	Dural incision	- easily sutured and watertight - no immunogenicity - flexible and easily suturable	(79–81)
Bovine and equine	Rabbit (*In vivo*)	Ophthalmic surgery (corneal perforations, glaucoma, etc.)	- safe and rapid - no conjunctival erosion followed - well-healing the corneal wounds - always available in contrast to human doner	(82–87)
Bovine	Human (*In vivo*)	Bony and periodontal defects	- resorbed without inflammation - stabilized the resorption process - significant improvement in regenerating the tissue	(88, 89)
Bovine	Human (*In vivo*)	Abdominal defects (ventral hernia, Urethral strictures)	- lowering the recurrence rate - no fistula development - complete reconstruction	(90, 91)

## 5 Artificial intelligence (AI) and machine learning (ML) intersection with decellularization in tissue engineering

AI and ML are the most significant approaches in the scope of tissue engineering particularly in areas of decellularization, protocol optimization, histological analysis as well as ECM characterization. These computational methods can provide high accuracy, Standardization in biomedical application with the lowest experimental cost (92).

### 5.1 AI in decellularization processes

Decellularization is used to preserve the ECM, allowing it to function as a framework for regeneration by removing cellular components from tissues. Meanwhile, the AI models that have excited researchers are especially good at identifying the underlying factors that affect different treatments. These models examine enzyme levels, blood flow rates through tissues and appreciations of how long someone is exposed to something. Supervised learning models in particular have been employed to optimize detergent formulations and assess tissue integrity following decellularization. Deep learning has also been employed to assess decellularization efficacy through imaging and chemical data, which dramatically reduces the need for costly trial-and-error experimentation (93). Moreover, the use of feedback systems allows for the adjustment of characteristics used in the decellularization process for real-time monitoring throughout the entire process, further enriching AI's role in decellularization. Predictive modeling can allow certain key structural components to remain preserved, minimize damage to the equipment, allow the consistency and integrity of the tissue not to be compromised, and will make them more viable for use in biomedical applications (94).

### 5.2 Optimization of decellularization protocols

Historically, decellularization protocols have involved a significant amount of trial and error to determine optimal conditions and really nail everything down. This enables the optimization of these protocols by way of AI sifting through massive datasets to determine the optimal parameters. One aspect of AI, reinforcement learning, can potentially be employed to dynamically change variables such as temperature, pressure, and exposure duration to enhance ECM preservation. Plus, AI simulations allow researchers to organize situations before entering lab work this allows them to predict what results will be and all but remove the use of materials or other assets. AI-driven computational fluid dynamics (CFD) models have been used to simulate perfusion decellularization, allowing for the identification of optimal flow rates and exposure times to achieve full cellular removal. These simulations are useful because they allow us to sort out protocols in a gentle way, without too much guess and check in the chemistry lab (7, 95).

### 5.3 AI for histological analysis and ECM characterization

While decellularized tissues have many potential applications, detailed tissue analysis is critical to determining the quality of the decellularized tissues. With the advent of AI-assisted image processing tools, automatic segmentation, classification, and quantification of tissue structures have recently transformed studies of the tissue. Convolutional neural networks (CNNs) excel at identifying residual cellular material and evaluating ECM quality. AI-assisted histological analysis provides consistency between different observers, thereby minimizing the possibility of subjective discrepancies in manual evaluations (96). AI is also instrumental in ECM characterization through the integration of various data sources, including biochemical assays, mechanical testing and microscopic imaging. This is where ML models come in handy, in correlating ECM composition to its functional properties. In other words, making sure decellularized scaffolds retain their mechanical strength and biochemical signatures that are conducive to transplantation (97, 98).

## 6 Pericardium commercial products

Products made from decellularized pericardium that are readily accessible on the market are frequently employed for soft tissue repair and rebuilding in a variety of surgical procedures. Regarding heart tissue repair and reconstruction as well as other cardiovascular and vascular surgeries, these products provide a secure and efficient solution. Peri-Guard®, produced by Synovis Life Technologies, is one of the most well-known products in this category. A decellularized pericardial patch called Peri-Guard® was created especially for repairing and reconstructing soft tissue. In surgical operations, it is frequently utilized to repair injured tissues and speed up healing (99). CardioCel®, made by Admedus in Malaga, Western Australia, is another noteworthy item. A decellularized bovine pericardial patch known as CardioCel® is frequently used in cardiovascular surgery. It is a vital tool for cardiac surgeons since it is very successful at mending and rebuilding heart tissues. Excellent biocompatibility and durability are provided by this product (100). Edwards Bovine Pericardial Patches are provided by Edwards Lifesciences, a company situated in Irvine, California, USA. These patches undergo decellularization to eliminate cellular matter and are also made from bovine pericardium. They are suitable for a variety of heart repair treatments due to their strength and flexibility, which are well known for them (101).

Equine pericardium serves as the raw material for Matrix PatchTM production by Auto Tissue Berlin GmbH. This decellularized patch is frequently used in cardiovascular procedures and has great handling qualities (102). No-React®, manufactured by BioIntegral Surgical, is made from decellularized pig pericardium. This product is renowned for its great biocompatibility and low immunogenicity (103). Peri-Guard by Baxter International Inc., with headquarters in Deerfield, Illinois, uses pericardium from cows. It goes through a process called GA-crosslinking to improve its toughness and mechanical qualities (104). Peripatch-EQ and Peripatch-BV are provided by Neovasc Inc., a Canadian company based in Richmond, British Columbia. These patches are made from the pericardium of horses and cows, respectively. Due to their good handling characteristics, they are frequently employed in cardiovascular procedures (105).

The pericardium of cows is used to make PhotoFix®, which is produced by CryoLife. Decellularization is used on this product to keep the ECM intact while removing cellular components. It is renowned for being flexible and simple to use (106). The pig pericardium used to make Vascutek pig Pericardial Patch is produced by Vascutek LTD., which has its headquarters in Inchinnan, UK. It receives a GA-crosslinking procedure to improve its mechanical qualities (107). Bovine pericardium is the source of the SJM BiocorTM Patch by St. Jude Medical, which has outstanding biocompatibility and longevity (108). Although it also uses bovine pericardium, the SJM Pericardial Patch with EnCapTM AC Technology by St. Jude Medical integrates cutting-edge technology for better tissue integration (109).

Both bovine and porcine pericardium are used in the production of SURGIFOC by FOC Medical in Buenos Aires, Argentina. It receives a GA-crosslinking procedure to improve its mechanical qualities (110). The Tissue Regenix Group PLC's dCELL® vascular patch, which is made from pig pericardium, has outstanding biocompatibility and improved tissue regeneration capacities (111).

Baxter's Vascu-Guard is made from bovine pericardium and offers superior handling and improved tissue integration features (112). Products made from decellularized pericardium that are sold commercially are essential for soft tissue restoration and repair during a variety of surgical procedures. In the realm of cardiovascular surgery, these tools give doctors secure and efficient ways to repair damaged tissues, improving patient outcomes (113, 114) (Table 3).

**Table 3:** Decellularized pericardium commercial products.

Product name	Manufacturers	Applications	Ref.
Peri-Guard®	Synovis Life Technologies	Skin	(99)
CardioCel®	Admedus in Malaga, Western Australia	Cardiac	(100)
Edwards Bovine Pericardial Patches	Edwards Lifesciences, Irvine, California, USA	Cardiovascular	(101)
Matrix PatchTM	Auto Tissue Berlin GmbH	Cardiac	(102)
No-React®	BioIntegral Surgical	Pulmonary valve	(103)
	Mississauga, Ontario, Canada		
OrthADAPT™	Synovis Orthopedic and Wound Care, USA	Reinforce soft tissue	(115)
Peripatch-EQ	Neovasc Inc.	Cardiovascular	(105)
Peripatch-BV	Richmond, British Columbia, Canada		
PhotoFix®	CryoLife	Cardiovascular	(106)
	Kennesaw, Georgia, USA		
Vascutek Porcine	Vascutek LTD	Aortic valve	(107)
Pericardial Patch	Inchinnan, UK	Cardiovascular	(116)
SJM Biocor^TM^ Patch	St. Jude Medical	Heart valve	(108)
	Saint Paul, Minnesota, USA		
SURGIFOC	FOC Medical	Cardiovascular	(110)
	Buenos Aires, Argentina		
Tutopatch®	RTI Surgical, Inc., USA	Bone implants	(117)
	Med & Care, Poland	Soft tissue grafts	
Tutoplast®	Innovative Ophthalmic Products, Inc., Costa Mesa, CA, USA	Soft tissue grafts	(83)
dCELL® vascular patch	Tissue Regenix Group PLC	Vascular	(111)
Vascu-Guard	Baxter	Vascular	(112)
Veritas®	Synovis Surgical Innovations, USA	Pelvic floor	(118)
		Soft tissue deficiencies	

## 7 Challenges, limitations, and future directions

Decellularized pericardium is so promising as a scaffold for tissue engineering applications. Before these techniques be widely used in clinical practice, there are several restrictions and difficulties that must be overcome. The short lifespan is one of the main drawbacks of decellularized pericardium in tissue engineering techniques. The long-term pressures and strains necessary for permanent tissue replacement may be beyond their ability to bear, even though they can offer temporary support. Additionally, using tissues taken from animals may cause the receiver to have an immunological reaction, which could result in rejection or other difficulties. Furthermore, there aren't enough clinical studies on the security and effectiveness of these treatments and using decellularized pericardium may not be appropriate for all tissue types or applications (15, 23, 119).

Another approach includes Determining successful tissue decellularization is challenging due to limitations in current evaluation methods. Traditional techniques, such as DNA quantification and histological staining, require tissue damage, hindering their use in practical applications. However, the traditional methods are time-consuming and requires constant attention. To overcome these obstacles, ML to precisely identify the completion of the decellularization process developed an innovative, automated system can be utilized. This system eliminates the need for tissue damage or continuous monitoring (120, 121).

Successful tissue engineering depends on achieving correct integration between the decellularized pericardium and host tissue yet fostering this integration can be difficult (36). Getting adequate cell seeding and integration into the decellularized pericardium is one of the main problems. Technically challenging tasks include stimulating cell adhesion, proliferation, and differentiation while ensuring uniform cell dispersion across the scaffold. Additionally, for nutrient delivery, waste elimination, and general tissue viability, significant vascularization is required. However, the decellularized pericardium's capacity to maintain long-term tissue life can be constrained by the difficulty of encouraging blood vessel ingrowth (35). Converting encouraging *in vitro* findings into clinical applications is another difficulty. Extensive preclinical research, proof of safety and efficacy in animal models, and ultimately human clinical trials are required to successfully translate this technology from laboratory research to clinical practice. Additionally, there may be difficulties in terms of time, resources, and adherence to safety regulations when scaling up the production of these patches to satisfy clinical demands and regulatory criteria (19).

Cost-effectiveness is another crucial factor. It is necessary to weigh prospective benefits in terms of patient outcomes and healthcare costs against the costs associated with development, production, and implementation. Additionally, it can be difficult to obtain enough viable aortic valve interstitial cells (AVICs) from both pig and human sources. It can be challenging to get reliable findings because of the variability in these cells’ availability and quality. For tissue engineering to be successful, it is also essential to guarantee the viability of AVICs while they are being seeded (16, 122).

Another difficulty is obtaining consistent results across several studies and samples. The technique's reproducibility may be impacted by variations in cell behavior, tissue quality, and other elements. Additionally, it could be challenging to compare the outcomes of various research due to the lack of established protocols for decellularization, recellularization, and assessment techniques. Finally, studies examining tissue engineering approaches based on decellularized pericardium have several disadvantages. These include small sample sizes, bias potential, and quick assessments. Furthermore, not all cellular residues from the decellularization procedure utilized to remove cellular components from the bovine pericardial patches may have been eliminated, which could possibly cause an immunological reaction in the recipient. Despite these difficulties, tissue engineering approaches based on decellularized pericardium show significant potential for enhancing patient outcomes in cardiac surgery and other applications. To thoroughly assess their potential advantages and hazards as well as to address the difficulties involved in implementing them in clinical practice, more study is required (71, 123–125).

In tissue engineering and regenerative medicine, a hotly debated question refers to the route that decellularized pericardium will take in the future. Decellularized pericardium has demonstrated a great potential as a scaffold for several procedures, such as conjunctival repair, vascular grafts, and tissue engineering of heart valves (71, 125, 126). Thousand possibilities to improve future of regenerative medicine, are now investigating in multiple area. One, is Enhancing the cell seeding procedure onto decellularized pericardium. When Amadeo et al. introduce the perfusion-assisted bioreactor technique, aortic valve cells seeding onto decellularized animal pericardium become more than a dream and open its way in the future (19). When contemplating the clinical use of decellularized pericardial patches, long-term healing results are critical. To understand the endurance and stability of decellularized bovine pericardial patches throughout time, Umashankar et al. examined the long-term healing of these patches (38). Santoro et al. also looked at the viability of utilizing aortic valve interstitial cells from pigs or humans on fixative-free decellularized animal pericardium. This study offers important insights on cell integration and compatibility with the scaffolding (16). Not only these revolutions could happen in cardiovascular surgery, but also hydrogels are the way to treat diseases, deliver drugs, heal wounds, etc. For all kind of problems from cardiovascular to musculoskeletal. Decellularized pericardial matrix gel in injectable form has also been created as prospective heart tissue engineering scaffolds (29). These injectable gels have benefits like minimally invasive delivery and capacity to conform to uneven heart shapes. Although decellularization methods are advance nowadays, but they are not perfect yet. Finding ways for decellularization that can improve mechanical characteristics, is one of the focus points. For instance, Kimicata et al. evaluated the usage of (polypropylene fumarate) biohybrid in combination with decellularized pericardial ECM for small-diameter vascular graft applications. The study's findings suggested its potential as a synthetic graft substitute by demonstrating superior mechanical characteristics and biocompatibility (32). This also can be followed by strategies that could encourage cell adhesion and growth, offering a viable remedy for ocular surface reconstruction. As showed by Crosslinked decellularized swine pericardium was investigated by Chen et al. as a substrate for conjunctival restoration (20). Additionally, in a pig carotid artery model, Chlupac et al. investigated vascular remodeling utilizing clinically used patches and recellularized decellularized pericardial matrices with autologous or allogeneic cells. According to the results, both autologous and allogeneic cell-seeded matrices exhibited favorable remodeling traits, pointing to their potential for use in vascular tissue engineering (39).

This article show how decellularized pericardium will be used in the future in a variety of biomedical domains, including cardiovascular tissue engineering and regenerative medicine. To fully realize the promise of this biomaterial, additional study is required to optimize production processes, improve mechanical qualities, increase long-term durability, and assess clinical outcomes.

## 8 Discussion

This review aimed to introduce and make the selection of decellularization methods simple for researchers by providing a complete list. But one question remains; which approach is the best? Surprisingly our answer is “we don’t know!” because it all depends on your study design, application, and purpose of your decellularized pericardium; you need better mechanical properties, better biocompatibility, better preservation of ECM or just it's important for you to cells completely being removed for your tissue? So, here we’re going to make it even more simple for you. Choosing the best approaches at first need a deep dive in the advantages and disadvantages of each method. Using Detergents (SDS, Triton X-100, SD, etc.) as one of the most common methods for decellularization have benefits like an effective removal of cells which make them to be used frequently and they are also inexpensive. But on the other side, they Can be harsh and potentially damage the ECM if not used carefully (about their concentration, timing and temperature) but when used correctly, they even can preserve collagen, elastin, and glycosaminoglycans (GAGs). And they may also require extensive washing to remove residual detergents that can damage our study model (especially important for the animal and human trials) (41, 127).

Another method for decellularization that were discussed was “enzymes” (DNase, RNase, PLA2, etc.). using enzymes to dissolve and remove exact components that we need, become popular within researchers to conduct exact experiments. These agents can effectively remove DNA and RNA, potentially preserving more delicate ECM components, so, it would be a great chose for situations that need the ECM to be finely preserved. But as an unwritten role, the more delicate work needs more money. They may also require optimization of enzyme concentration and incubation time which need an exact expert researcher (128, 129). One of the first methods that researchers find out that it could work; Physical Methods [High Hydrostatic Pressure (HHP), Freeze-Thawing, etc.]. they potentially have less damage to ECM compared to chemicals. But they require specialized equipment and, they may not be as effective in complete cell removal for thicker tissues, and they need other methods by their side (15, 41). Finally combined methods which discussed completely. Based on the agents that each methods contain they could have various pros and cons, but on the overall view, they can enhance decellularization efficacy while minimizing damage to the ECM, as the purpose of these methods (32).

As mentioned in Table 3, pericardium has been used as a suitable ECM source in repair, especially in cardiovascular tissues. Choosing the right pericardium source can have a great impact on the mechanical, biological, and functional properties of the final scaffold, which may also vary its application. Human pericardium is the best choice due to its structural similarity and high biocompatibility, but the challenges in using this tissue have limited its application, including limited availability compared to animal sources. However, despite the decellularization process in this tissue, it can be suitable for human grafts (130, 131). Bovine pericardium is one of the most widely used sources in the fabrication of heart valves and other tissue engineering scaffolds. The dense collagen structure increases resistance to mechanical stresses. However, it requires optimization processes in the decellularization process to reduce immunogenicity. Porcine pericardium is more like human pericardium and has found widespread use in tissue engineering. Due to the fibrous arrangement of collagen, it is more flexible than bovine pericardium (132, 133). Some studies have also reported interesting results comparing equine and bovine pericardium, pointing to the better use of equine pericardium because it is more flexible in cardiac applications (134).

Based on these comparisons we could conclude some point that may help researcher to choose the best method based on their work.

• **For preserving mechanical properties while achieving decellularization:** HHP combined with mild chemical treatments appears promising. HHP can disrupt cells while causing less damage to the ECM than harsh chemicals.
• **For effective removal of cellular components**: A combination of detergents like SDS and TX is highly effective.
• **For cardiovascular applications:** The TRITDOC method has been developed as a holistic approach to heart valve tissue engineering with a focus on ECM preservation and removal of cellular components and is a more advanced method compared to just using SDS/TX. Perfusion-assisted bioreactors are also very effective in seeding cells into decellularized scaffolds in cardiovascular applications.
• **For applications where toxicity is a concern:** Freeze-thawing is a good option. This is because it avoids the use of chemical agents.
• **For applications requiring preservation of delicate ECM components or where harsh chemicals are a concern:** methods involving enzymes or physical methods (or a combination) may be preferred.

## Conclusion

Tissue engineering's investigation of decellularized pericardium has opened exciting new possibilities for the creation of cutting-edge biomedical products. Decellularized pericardium has several qualities that make it an attractive option for tissue engineering applications, including biocompatibility and biomechanical integrity. We highlight its efficaciousness in promoting cell adhesion, proliferation, and differentiation, thereby aiding in the development of functional tissue constructs. Even with the significant advancements this analysis points out, we nevertheless need to recognize the information gaps that remain. Subsequent investigations ought to concentrate on streamlining decellularization processes, improving scaffold characteristics, and investigating novel approaches to augment integration with host tissues. To confirm the safety and effectiveness of decellularized pericardium-based constructions in practical applications, including long-term biocompatibility tests and clinical trials research are necessary. In conclusion, the use of decellularized pericardium in tissue engineering is a rapidly developing field that has the potential to revolutionize regenerative medicine in near future with further research.
